# A Modified Langmuir Model for Moisture Diffusion in UGFRE of Composite Insulator Considering the Composite Degradation

**DOI:** 10.3390/polym14142922

**Published:** 2022-07-19

**Authors:** Zhikang Yuan, Cheng Wang, Lijun Jin, Youping Tu, Yingyao Zhang, Zhenlian An, Yongfei Zhao

**Affiliations:** 1 Department of Electrical Engineering, College of Electronic and Information Engineering, Tongji University, Shanghai 201804, China; zhikangyuan@tongji.edu.cn (Z.Y.); zhangyingyao@tongji.edu.cn (Y.Z.); zan@tongji.edu.cn (Z.A.); 2 State Key Laboratory of Alternate Electrical Power System with Renewable Energy Sources, School of Electrical and Electronic Engineering, North China Electric Power University, Beijing 102206, China; typ@ncepu.edu.cn (Y.T.); zyf534503401@163.com (Y.Z.); 3 State Grid Hunan Extra High Voltage Substation Company, Changsha 410004, China; wangcheng9506@163.com

**Keywords:** modified Langmuir model, moisture diffusion, unidirectional glass fiber reinforced epoxy resin (UGFRE), composite degradation

## Abstract

Water invasion induced aging and degradation of the unidirectional glass fiber reinforced epoxy resin (UGFRE) rod is inferred as the primary reason for the decay-like fracture of the composite insulator. In this paper, the moisture diffusion processes in the UGFRE from different directions at various test humidities and temperatures are studied. The moisture diffusion of the UGFRE sample obeys the Langmuir diffusion law under the humidity conditions of 53%, 82% and 100% at 40 °C. In deionized water, the moisture diffusion of the UGFRE sample also obeys the Langmuir diffusion law when the invading direction is vertical to the glass fiber. However, when the water invades the UGFRE sample, parallel with the glass fiber, the weight loss caused by composite degradation should not be neglected. A modified Langmuir model, taking Arrhenius Theory and the nonlinear aging characteristic of the composite into consideration, is proposed and can successfully describe the moisture diffusion process. Both the glass fibers and epoxy resin will degrade in the deionized water. The glass fibers show better resistance to degradation than the epoxy resin. The epoxy resin degrades from the glass fiber/epoxy resin interface and become fragments. For composite insulators, the water invasion through the ends should be avoided as far as possible, or the degradation of the UGFRE rod will result in decay-like fracture.

## 1. Introduction

Glass fiber reinforced polymers (GFRP) are widely used in the areas of new energy, aerospace, nuclear fusion, construction, and power transmission system for the good electric insulation performance, high mechanical strength, light weight and low cost [1,2,3,4,5]. In extra-high voltage (EHV) and ultra-high voltage (UHV) transmission lines, unidirectional glass fiber reinforced epoxy resin (UGFRE) rod performs as the internal insulation in composite insulator, which is the key equipment to connect the high voltage wire and tower. In recent years, a new type of failure named decay-like fracture of composite insulator, which would lead to the drop of high voltage wire and threaten the lives near the tower, has drawn high attention from academia and industry. In the reported cases, the UGFRE rods in the composite insulators degrade like decay woods and fracture at the high voltage end [6,7]. Water invasion induced aging and degradation of UGFRE is inferred as the primary reason for the decay-like fracture [7,8,9]. However, moisture diffusion process in the UGFRE rod of composite insulator remains unclear.

In composite insulator, previous work always cares about the moisture diffusion process in silicone rubber [10,11,12], which performs as the external insulation, and the interface between silicone rubber housing and UGFRE rod [12,13,14]. With the increasing number of fracture accidents of composite insulator, especially in the areas of high ambient humidity, moisture diffusion in the UGFRE rod has attracted attention. Liang et al. studied the diffusion processes of deionized water, NaCl solution, and nitric acid solution, respectively, in the plate-shaped UGFRE samples. In deionized water and NaCl solution, the GFRP weight increased as the liquid invaded. In the nitric acid solution with the concentration of more than 0.1 mol/L, it was found that the UGFRE weight decreased with the diffusion time. The dissolution of the corroded epoxy resin matrix and ion depletion of glass fiber in the nitric acid solution lead to the weight change directly [15]. Tu et al. put cylindrical UGFRE samples in deionized water for hygrothermal aging. It was found that the moisture absorption ability of the UGFRE enhanced with the aging time, which would lead the temperature rise of UGFRE rod under AC and DC electric fields [16]. The electric specialists pay more attention on the consequences of the UGFRE after liquids invasion. However, the moisture diffusion process, which can reveal the decay-like degradation process of UGFRE rod in composite insulator, also needs further exploring.

As for the moisture diffusion process, Kumosa et al. carried out the moisture absorption tests on the unidirectional glass/polymer composite materials, based on E-glass, high seed count ECR-glass and low seed count ECR-glass fibers with modified polyester, epoxy and vinyl ester resins. It was found that the epoxy-based materials did not reach equilibrium and kept slowly taking on more moisture in a non-Fickian manner [17]. Joliff et al. found that the evolution of composite absorption was better described by Langmuir than Fick model. Modified water diffusion kinetics in unidirectional glass fiber composite considering the interphase was proposed [18]. Measurement of weight change is the method to obtain the moisture absorption in the composite and Langmuir model shows promising prospects on describing the moisture diffusion process. However, there will be weight loss caused by degradation of the composite during long-term moisture absorption test. There is still no model that can describe the moisture diffusion process considering the composite degradation.

In this paper, moisture absorption tests are carried out on UGFRE samples, which are cut off from the core rod of composite insulator. Moisture diffusion in the UGFRE samples from different directions at various test humidities and temperatures is obtained by weight measurement. A modified Langmuir model considering the composite degradation is proposed and the moisture diffusion process is discussed.

## 2. Samples and Experiment

### 2.1. Samples

**Figure 1:**
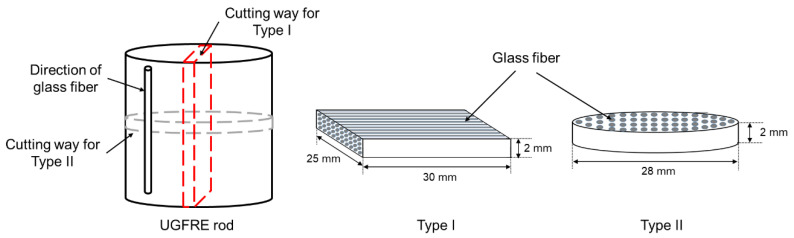
Obtaining methods and schematic diagrams of sample Type I and Type II.

**Figure 2:**
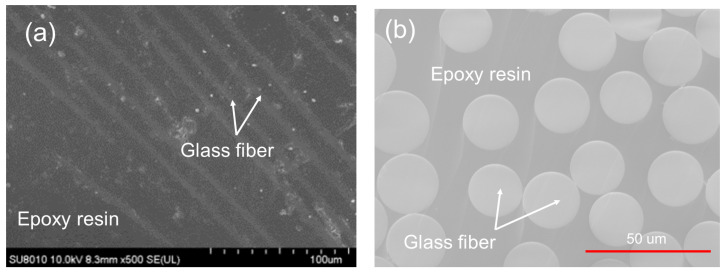
Surface micro morphology of the sample. (**a**) Type I and (**b**) Type II.

**Figure 3:**
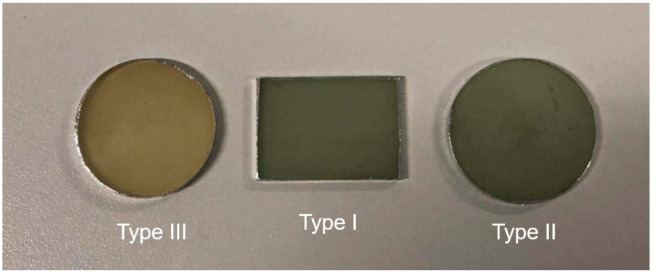
The appearance of the three types of samples.

In order to study the moisture diffusion process in UGFRE from different directions, two types of samples are prepared. The samples are from the UGFRE rod in composite insulator. The UGFRE rod is manufactured by pultrusion process and the mass ratio of boron-free glass fiber and bisphenol-A (E51) epoxy resin is about 8:2. The curing agent is methyl tetrahydrophthalic anhydride (MTHPA). The diameter of the glass fiber is about 20 μm. Sample Type I is cut off along the axial direction of the UGFRE rod, as the red dotted box in Figure 1 shows, and a cuboid sample of 30 mm × 25 mm × 2 mm is obtained. The direction of the glass fiber is parallel to the cutting plane. Sample Type II is cut off along the radial direction of the UGFRE rod, as the gray dotted box in Figure 1 shows. A cylinder sample with 28-mm diameter and 2-mm thickness is obtained and the glass fiber is vertical to the cutting plane. The surface microstructures of the sample Type I and Type II are obtained by Hitachi SU8010 are shown in Figure 2. It can be found that glass fibers distribute uniformly in UGFRE rod and the interface between the glass fiber and epoxy resin matrix is constructed well in Figure 2a. In Figure 2b, all the glass fibers appear intact and are covered well by epoxy resin. Sample Type III, epoxy resin with no glass fiber inside, is set as the control group. The sample shows the same size as Sample Type II. The appearance of the three types of samples are shown in Figure 3.

### 2.2. Experiment

The humidity of test environment is constructed by using saturated salt solution according to ISO 483 [19]. Three humidity conditions, 53%, 82% and 100%, are constructed by the saturated solution of NaBr, KCl and deionized water, respectively, at 40 °C. As is known, composite insulators operate in outdoor environment. Considering that 40 °C is the highest temperature in summer that the composite insulator may face in operation, we choose the temperature of 40 °C to do the experiments. Before the test, one third of a 27 cm × 21 cm × 20 cm airtight box is filled by the solution at first. After 2 h, it reaches to the saturated humidity in the box. Then, the samples are placed on a plate supporter, which is 5 cm above the solution surface, in the box. There is no direct contact between the samples and the solution. The other condition is that the samples are immersed in the deionized water. Three other airtight boxes are prepared to study the moisture diffusion at different temperatures. The airtight boxes are placed in the chambers with constant temperatures of 40 °C, 60 °C and 80 °C, respectively. All the test conditions are shown in Table 1. In order to get rid of the effect of moisture diffusion from the side on the test results, aluminum foil paper is glued to the edge of the samples before the test.

To obtain the moisture diffusion process, a BSM120.4 electronic analytical balance with the test precision of 0.1 mg, supplied by Shanghai Jingzhuo Electronic Technology Co., Ltd. is employed to measure the weight change of the sample. Before weighting, the samples are moved out of the box and the excess water on the surface of the sample is removed. The weighting process is finished within 2 min. The weight change ratio *m*(*t*) at time *t* can be obtained using Equation (1),

(1) $$m(t)=\frac{m_{t}-m_{0}}{m_{0}}\times 100\%$$

where *m_t_* is the weight of the UGFRE sample at time *t* and *m*_0_ represents the initial weight of the dry sample. Three samples of each type are prepared and measured. The average weight change ratio and the standard deviation of the three samples are recorded as the test results.

## 3. Modified Langmuir Model Considering Composite Degradation

According to Langmuir model, there are two states of water molecule, free water molecule and bound water molecule, in the glass fiber reinforced composite [20]. Free water molecules can move freely in the molecular gap and bound water molecules are absorbed on the micro-surface of the composite. These two states of water molecule keep converting to each other. The moisture diffusion process can be described by following Equation (2), which is proposed by Carter and Kibler [21],

(2) $$\left\{ \begin{array}{l} \frac{\partial u_{f}}{\partial t}=D_{f}\frac{\partial^{2}u_{f}}{\partial x^{2}}-\frac{\partial u_{b}}{\partial t}\\ \frac{\partial u_{b}}{\partial t}=\alpha u_{f}-\beta u_{b} \end{array}\right.$$

where *u_f_* and *u_b_* are the concentrations of free water molecule and bound water molecule, respectively. *α* is the bonding coefficient, which describes the converting probability from free water molecules to bound water molecules. *β* is the de-bonding coefficient, which describes the converting probability from bound water molecules to free water molecules. *D_f_* is the diffusion coefficient of the free water molecule. The diffusion reaches saturation when the converting between free and bound molecular water is in dynamic equilibrium, as shown in Equation (3),

(3) $$\frac{\partial u_{b}}{\partial t_{\infty }}=\alpha u_{f\infty }-\beta u_{b\infty }\;=\;0$$

where *u_f_*_∞_ and *u_b_*_∞_ are the concentrations of free water molecule and bound water molecule, respectively, when the diffusion reaches saturation.

The relationship between the relative moisture content in the sample *m*(*t*)/*m_∞_* and diffusion time can be obtained by solving the partial differential equations above and the result is shown in Equation (4), where *y*(*t*) is shown in Equation (5).

(4) $$\begin{array}{ll} \frac{m(t)}{m_{\infty }} & =\frac{\beta }{\alpha +\beta }\exp\left(-\alpha t\right)y\left(t\right)+1-\exp\left(-\beta t\right)\\ & +\frac{\beta }{\alpha +\beta }\left[\exp\left(-\beta t\right)-\exp\left(-\alpha t\right)\right] \end{array}$$

(5) $$y\left(t\right)=1-\frac{8}{\pi^{2}}\sum_{n=0}^{\infty }\frac{1}{\left(2n+1\right)}\exp\left[-\frac{D{\left(2n+1\right)}^{2}\pi^{2}t}{{\left(2h\right)}^{2}}\right]$$

Considering that there will be weight loss caused by composite degradation during long-term moisture absorption test, a modified factor *M*(*t*)/*m_∞_*, which is related to the test time *t* and temperature *T*, is proposed, as shown in Equation (6),

(6) $$\frac{M(t)}{m_{\infty }}{=2}^{\frac{\left(T_{ref}-T\right)}{10}}[\exp(k_{ref}t)-C_{0}]$$

where *M*(*t*)/*m_∞_* is the relative weight loss caused by the dissolution of the corroded epoxy resin matrix and ion depletion of glass fiber. *T* is the test temperature. *T_ref_* is the reference temperature. *k_ref_* is the reaction constant at the reference temperature. *C*_0_ is a constant, *C*_0_ = 1, to ensure that *M*(0)/*m_∞_ =* 0 when *t* = 0. According to Arrhenius Theory, when the temperature increases by 10 K, the rate of chemical reaction and physical dissolution is doubled, and the service life of the material is halved [22]. So the exponential term 2^(*Tref − T*)/10^ is constructed. The nonlinear aging characteristic of the composite is also taken into consideration. The degradation and dissolution rate will increase with the aging time. The exponential term based on *e*, exp(*k_ref_t*), is used to describe the nonlinear process of aging [23,24]. After adding *M*(*t*)/*m_∞_* in the traditional Langmuir model, the modified Langmuir model considering composite degradation is shown in Equation (7).

(7) $$\begin{array}{ll} \frac{m(t)}{m_{\infty }} & =\frac{\beta }{\alpha +\beta }\exp\left(-\alpha t\right)y\left(t\right)+1-\exp\left(-\beta t\right)\\ & +\frac{\beta }{\alpha +\beta }\left[\exp\left(-\beta t\right)-\exp\left(-\alpha t\right)\right]-2^{\frac{\left(T_{ref}-T\right)}{10}}[\exp(k_{ref}t)-C_{0}] \end{array}$$

## 4. Results

**Figure 4:**
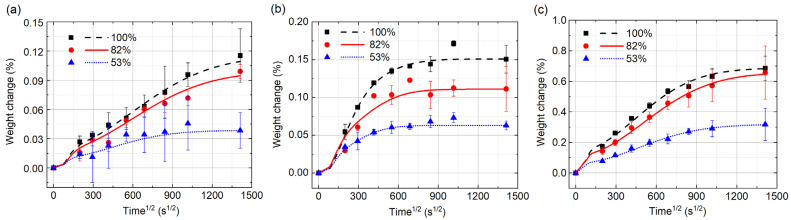
Weight changes of the samples under different environment humidities at 40 °C. (**a**) Type I; (**b**) Type II; (**c**) Type III.

Figure 4a–c show the weight changes of the samples, Type I, Type II and Type III, under different ambient humidities, 53%, 82% and 100%, respectively. The standard deviations are also shown in the figures. The weights of all the samples increase with the test time. The higher the ambient humidity is, the higher rate of the weight increase will be. Langmuir model is used to fit the data points of weight change. The fitting results are in good agreement with the data points, as shown in the figures, which indicates that the moisture diffusion in UGFRE obeys Langmuir diffusion law under the chosen ambient humidities. Then, the moisture diffusion process and the saturated moisture content in the three types of samples are put into comparison. In Figure 4a, the samples of Type I show the lowest moisture absorption rate, longest time to reach the saturated state and least content of saturated moisture absorption. After 554 h’ moisture absorption, the moisture contents are only 0.115%, 0.099% and 0.038%, respectively, in the ambient humidities of 100%, 82% and 53%. Compared to the epoxy resin, the moisture absorption ability of the glass fiber is extremely weak. The glass fibers on the surface of the sample, as shown in Figure 2a, prevent the moisture from invading into the UGFRE by the direction which is vertical to the glass fiber. That’s how the lowest moisture absorption rate of Type I comes. On the other hand, due to the random distribution of the glass fibers on the surface, the area of the epoxy resin, which is exposed to the deionized water, differs from sample to sample. That’s how the large deviation for Type I sample comes. The samples of type II show the highest moisture absorption rate, shortest time to reach the saturated state, as shown in Figure 4b. The content of saturated moisture absorption is less than the samples of Type III. With the increase of the humidity, the saturated moisture content and the time to reach the saturated state also increase. From Figure 2b, it is found that the glass fibers on the sample surface also reduce the effective area for moisture invasion. However, the interfaces between the glass fibers and the epoxy resin expose to the moisture directly, which supply the new channels for moisture to invade. That’s why the moisture absorption rate of Type II is the highest. On the other hand, most of the volume in the samples of Type I and Type II is filled by glass fibers, which leads to the lower saturated moisture content of Type I and Type II than that of Type III in Figure 4c.

**Figure 5:**
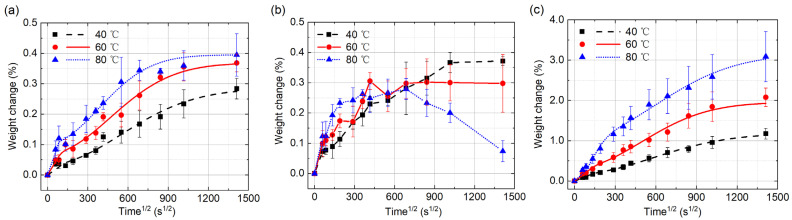
Weight changes of the samples in deionized water under different temperatures. (**a**) Type I; (**b**) Type II; (**c**) Type III.

The results above show that the moisture diffusion in UGFRE obeys Langmuir diffusion law under the ambient humidities of 53%, 82% and 100% at 40 °C. Figure 5a–c display the weight changes of the samples, Type I, Type II and Type III, in deionized water under different temperatures, 40 °C, 60 °C and 80 °C, respectively. In the samples of Type I, the invasion of the free water molecules plays the dominant role at the beginning and the weight increases sharply, especially at 60 °C and 80 °C, as shown in Figure 5a. Then, the moisture absorption rate slows down gradually till saturation. The saturated moisture content in the sample also increase with the temperature, reaching 0.284%, 0.368% and 0.395% at 40 °C, 60 °C and 80 °C, respectively. In the samples of Type III, the saturated moisture content is much higher, as shown in Figure 5c, reaching 1.176%, 2.078% and 3.089% at 40 °C, 60 °C and 80 °C, respectively. The data points in Figure 5a,c can be successfully fitted by Langmuir model, which means that the water diffusion processes in the samples of Type I and Type III also obey Langmuir diffusion law. However, in Figure 5b, the weight change of the samples cannot be fitted by Langmuir diffusion law. After 130 h in the 80 °C deionized water, the weight of the sample begins to decrease rapidly. The gained weight is only 0.074% at the 554th hour. At 60 °C, the weight of the sample increases to the highest, 0.306%, and remains stable after 48 h. At 40 °C, the weight of the sample keeps increasing gradually. All the data points in Figure 5b are connected by straight lines.

**Figure 6:**
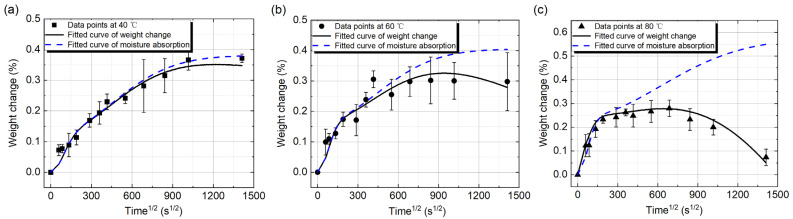
Weight changes of the samples fitted by the modified Langmuir model. (**a**) 40 °C; (**b**) 60 °C; (**c**) 80 °C.

In order to fit the data points in Figure 5b appropriately, the degradation of the Type II samples is taken into consideration. The modified Langmuir model, as shown in Equation (7), is used to fit the data points. We set 353.15 K, which equals to 80 °C, as the reference temperature at first. So the weight loss rates, which are induced by the degradation of the samples, at 313.15 K (40 °C) and 333.15 K (60 °C) are 1/16 and 1/4 of that at 353.15 K according to Equation (7). Then, *k_ref_* is obtained in this equation by curve fitting, *k_ref_* = 9.0 × 10^−6^. The fitted curves at 40 °C, 60 °C, and 80 °C are shown in Figure 6a–c, respectively. It should be noted that there are two fitted curves in each figure. The black solid line is the weight change of the sample. which is obtained by Equation (7) directly. and the blue dotted line is the content of moisture absorption in the sample, which is obtained by the first four terms in Equation (7). The consistent change of the data points and the fitted curve illustrates that the modified Langmuir model can successfully describe the weight change of the sample considering the composite degradation. In Figure 6a, the two fitted curves are almost overlapped for the first 100 h. Then, the degradation induced weight loss of the sample leads to the difference of these two curves. The curve of moisture absorption still obeys the Langmuir diffusion law. The weight decrease caused by the composite degradation becomes more and more with the test time. With the increase of the experimental temperature, it is found that the overlapped time of the two curves becomes shorter and shorter, as shown in Figure 6b,c, and the weight difference between the two curves becomes more and more at the same time. The reason is that the higher experimental temperature accelerates the physical or chemical degradation of the samples.

## 5. Discussion

**Figure 7:**
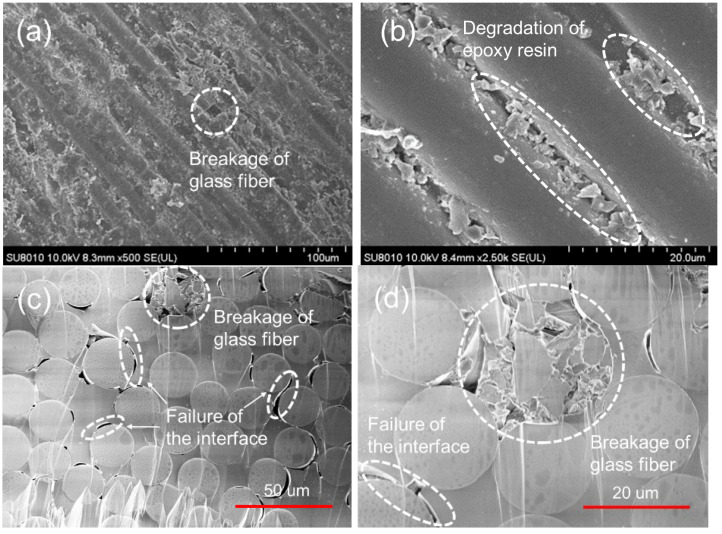
The micro morphology of the Type II sample after 480 h in the deionized water. (**a**) side view, magnified 500 times; (**b**) side view, magnified 2500 times; (**c**) cross section view, magnified 500 times; (**d**) cross section view, magnified 2500 times.

From the experimental results above, it can be concluded that the degradation of the composite leads to the weight loss during moisture absorption. To explore the degradation of the samples in micro scale, Hitachi SU8010 scanning electron microscope (SEM) is employed to obtain the micro morphology of the Type II sample. The results are shown in Figure 7. After immersing in the deionized water for 480 h, the side views of the sample, as shown in Figure 7a,b, display that the surface turns from smooth, in Figure 2a, to rough, in Figure 7a. Most of the glass fibers are intact, but the breakage of a glass fiber can also be found. Figure 7b shows the details of Figure 7a. The epoxy resin near the glass fibers has degraded into fragments and make the surface look rough. The degradation of the epoxy resin also leads to the exposure of the glass fibers to the water. On the cross section of the sample, as shown in Figure 7c, breakage of a glass fiber can be found while the other glass fibers remain intact, which is in similar with that in Figure 7a. Near the intact glass fibers, there are a lot of slim holes, which are caused by the degradation of epoxy resin, indicating that the degradation of the epoxy resin starts from the interface between the glass fiber and epoxy resin. In Figure 7d, the part of Figure 7c is magnified. The glass fiber is broken into pieces and the interface failure leads to the separation of the epoxy resin from the glass fiber. The SEM results illustrate that both the glass fibers and epoxy resin will degrade in the deionized water but the degradation degree differs. The glass fibers show better resistance to degradation than the epoxy resin that only a few glass fibers are found broken in the deionized water for 480 h while the epoxy resin degrades from the glass fiber/epoxy resin interface and become fragments.

As is known, temperature and humidity are two key factors that influence the degradation of composite [16,25,26]. In the experiments of Figure 4, the weight change of all the UGFRE samples obeys the Langmuir diffusion law under the humidity conditions of 53%, 82% and 100% at 40 °C. It doesn’t mean that there is no degradation of the UGFRE samples, but the degradation of the composite is not severe enough to result in the weight loss that can be measured by the balance. When the UGFRE samples are immersed in the deionized water, the invading way of the water is another factor that influence the degradation rate. In Type I samples, the sides of the glass fibers, which are covered well by the epoxy resin, are exposed to the water. Due to the weak water absorption ability, the glass fibers are barriers that can prevent water from invading. In type II samples, the water invades the composite through the glass fiber/epoxy resin interface. The defects of nanoscale in the interface can not only accelerate the water diffusion rate in the composite by capillarity [27], but also reserve the invaded water in the interface, which accelerates the degradation of the epoxy resin at the interface. That’s why the degradation of the epoxy resin starts from the interface. Compared to Type III, the interface between the epoxy resin and glass fiber enlarge the contact area between the sample and the deionized water dozens or even hundreds times of that of pure epoxy resin. The larger contact area will inevitably lead to the severer degradation of the samples at the same test condition. That’s why the degradation of Type II is much severer than Type III. For composite insulators, it is noted that the water invasion through the ends should be avoided as far as possible, or the degradation of the UGFRE rod will be inevitable, which will lead to abnormal temperature rise or even decay-like fracture.

## 6. Conclusions

In this paper, the moisture diffusion processes in the UGFRE, the core rod of composite insulator, from different directions at various test humidities and temperatures are studied. A modified Langmuir model to describe moisture diffusion in UGFRE considering the composite degradation is proposed. The conclusions are shown as follows.

1. The moisture diffusion of the UGFRE sample obeys the Langmuir diffusion law under the humidity conditions of 53%, 82% and 100% at 40 °C. With the increase of the humidity, the saturated moisture content and the time to reach the saturated state of UGFRE also increase. The glass fibers on the surface prevent the moisture from invading into the UGFRE sample by the direction which is vertical to the glass fiber. On the other hand, when the moisture invades the UGFRE sample parallelly with the glass fiber, the interfaces between the glass fibers and the epoxy resin will supply the new invading channels for moisture and accelerate the moisture absorption rate.
2. In deionized water, the moisture diffusion of the UGFRE sample also obeys the Langmuir diffusion law when the invading direction is vertical to the glass fiber. However, when the water invades the UGFRE sample parallelly with the glass fiber, the weight loss caused by composite degradation should not be neglected. The modified Langmuir model, taking Arrhenius Theory and the nonlinear aging characteristic of the composite into consideration, is proposed and can successfully describe the water diffusion process.
3. Both the glass fibers and epoxy resin will degrade in the deionized water but the degradation degree differs. The glass fibers show better resistance to degradation than the epoxy resin. The epoxy resin degrades from the glass fiber/epoxy resin interface and become fragments. For composite insulators, it is noted that the water invasion through the ends should be avoided as far as possible, or the degradation of the UGFRE core will be inevitable, which will lead to abnormal temperature rise or even decay-like fracture.

## Figures and Tables

**Table 1:** The test conditions.

Temperature	Test Humidity			
	53%	82%	100%	In Deionized Water
40 °C	√	√	√	√
60 °C				√
80 °C				√
